# Response feature analyses for repeated measures behavioral data

**DOI:** 10.1242/bio.062488

**Published:** 2026-08-05

**Authors:** Daniel T. Burns, Ken G. Gerow

**Affiliations:** Department of Mathematics and Statistics, University of Wyoming, Laramie, Wyoming 82071, USA

**Keywords:** Repeated measures, Behavior, Functional recovery, Spinal cord injury, Longitudinal data analysis, Response feature analysis

## Abstract

Repeated measures designs are a common experimental setup in biology, from ecology to biomedical sciences. Traditional statistical techniques for analyzing such data, such as repeated measures ANOVA (RMANOVA), have limitations that prevent them from being used to answer many questions relevant to measurements taken over time. Response feature analysis (RFA) techniques can provide an ideal means for answering those questions by analyzing data based on a creatively chosen summary function of the individual subject's data. The process and rationale for the RFA is laid out in a step-by-step manner and is demonstrated on an example of behavioral data from a rodent spinal cord injury study. In the original analysis, a difference in average functional score was only found at one timepoint early in the study. Using RFA, we show that the female rats reached 90% of their peak recovery 15.3 days (95% CI 1.73, 28.86 days) earlier than the male rats. RFA enables answering the important question ‘do the two sexes differ in the speed at which they recover functional ability?’, and, by way of demonstration, illustrates its flexibility in answering questions that traditional repeated measures analyses cannot.

## Teaser

**Summary:** Response feature analysis for functional behavioral data overcomes limitations of repeated-measures ANOVA, enabling flexible summaries of longitudinal data and revealing differences missed by traditional timepoint-based analyses.

## INTRODUCTION

Many biological studies employ designs that follow subjects across time. In ecology, studies have investigated acclimatization of translocated big horn sheep, mule deer movement, and waste output as a function of dietary inputs (Clapp et al., 2014; Hlucny et al., 2021; Sawyer et al., 2006) while studies in the biomedical sciences have looked into the effects of small-molecule screening in functional recovery following spinal cord injury (SCI), bacterial enzyme therapies for enhanced functional recovery in SCI, and sex differences in animal models of anxiety-like behavior (Chen et al., 2018; Mountney et al., 2013; Scholl et al., 2019).

Repeated measures ANOVA (RMANOVA) has long been a widely applied statistical technique for the analysis longitudinal data. Despite its widespread use, ANOVA has several limitations and shortcomings, for example: standard ANOVA models do not accommodate continuous covariates (Schober and Vetter, 2018); time, as a variable, can only be considered as a categorical variable, not continuous (van Wely, 2024); ANOVA assumes each individual is measured at the same time and does not allow for unequally spaced time intervals for different subjects (Schober and Vetter, 2018); and if a single observation is missing from a subject then the entire subject is excluded from the analysis (Everitt, 1998; Muhammad, 2023). This ‘complete case’ requirement is only valid if the values are missing completely at random (MCAR), and even if they are, this requirement can be very inefficient and lead to seriously biased and misleading results (Everitt, 1998).

Other analytical methods commonly used for longitudinal data include mixed-effects models and growth curve analysis, among others. Both methods are flexible when it comes to the inclusion of partially missing data, unequally spaced time points, and describing nonlinear relationships across time (Curran et al., 2010; Krueger and Tian, 2004). These methods, however, also have their own set of limitations to be aware of. Mixed-effects models can suffer from biased estimates and model degeneracy if there are not enough levels of a random effect (e.g. fitting sex as a random effect) (Silk et al., 2020), cannot model negative correlations within clusters, leading to deflated Type-I errors and invalid standard errors (Nielsen et al., 2021), and can be quite challenging to properly use and interpret (Bolker et al., 2009). Growth modeling requires data from at least three time points, recommended minimum sample sizes of 100, and difficulties in determining model fit (Boscardin et al., 2022; Xitao and Xiaotao, 2005). Another shortcoming of these methods is that they all, either implicitly or explicitly, treat time as an explanatory variable to control for, not as an outcome variable to be examined and compared.

Standard analytic approaches for repeated measures data have been around for a long time; Brian Everitt was a prominent exponent of such approaches (Everitt, 1995, 2002; Everitt and Pickles, 2000). Tucked away, almost hidden (and certainly unheralded) in that literature, is a method called response feature analyses (RFA). Everitt and Pickles (2000) elucidate methods for repeated measures analyses (RMA). As such, they briefly mention a ‘summary measures approach’ (RFA) but then move right into the details of RMA. In another such instance, Ramsey and Schafer (2012), in their chapter on RMA do the same thing: they point out that the most practical strategy is a univariate analysis on a summary measure (Section 16.2.3), but then move on to RMA, which is the topic of that chapter. RMA has its place and can be a powerful tool, but the summary measures approach (RFA) gets dismissed with little discussion. Gerow et al. (2021) has since exemplified the RFA method in the setting of some ecological examples. A main advantage to the approach of using summary statistics for repeated measures data is that it is does not sacrifice validity for the large gains in simplicity, and both the methods and results can be easily understood by those with a limited statistical background (Schober and Vetter, 2018).

Here, we demonstrate an application of the RFA method in an animal study-based biomedical example. We first briefly sketch the experimental design of Ghnenis et al. (2020), with a particular emphasis on the Basso-Beattie-Bresnahan (BBB) locomotor assessment of hindlimb motor recovery. We then demonstrate the power and flexibility of RFA to answer the question ‘Is there a difference in the time it takes to reach a certain recovery threshold and, if so, what is it?’, which standard repeated measures analyses like those mentioned above cannot answer (at least, not easily), using data from the BBB assessment administered in the study. By using these previously published data as an illustrative example, we show the ease and versatility with which RFA can be used and one approach for employing it.

## RESULTS

**Fig. 1.**
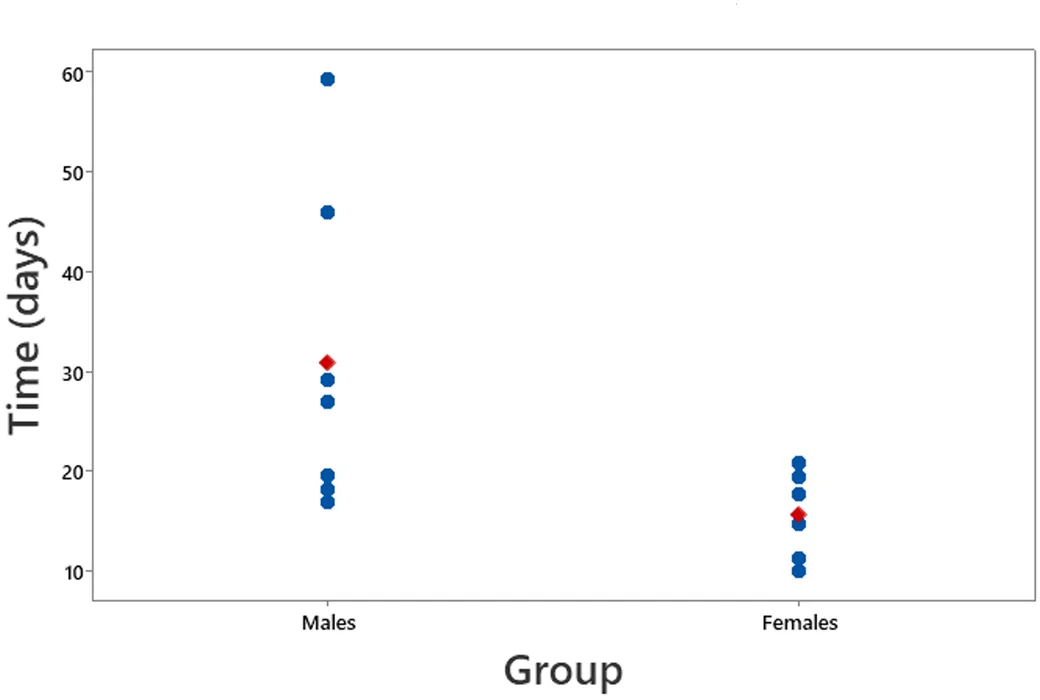
**Estimated time to 90% recovery for the 14 included animals.** Estimated times to ‘essential recovery’ for study animals. Sample means (diamonds) are 30.8 days (s.e.=6.0 days) for males (*n*=7) and 15.54 days (s.e.=1.5 days) for females (*n*=7).

The estimated times from the creative summary step, shown in Fig. 1, show the distinct separation in times for the two groups. On average, males took 30.8 days (s.e.=6.0 days) to reach 90% recovery while females only took 15.54 days (s.e.=1.5 days). A two-sample *t*-test yielded *P*=0.03 for the observed 15.3-day difference (95% CI 1.73, 28.86 days). These results also yield a very large effect size (Cohen's *d*=1.31) (Sullivan and Feinn, 2012). Given the relatively small sample sizes used here, we checked whether an outlier could shift the estimate. We introduced a fake 8th male (not included in the manuscript); its value would have to be 15.06 days or lower for the CI to include zero. We note that 15.06 is lower than the lowest observed male value (17). There are, however, four of seven female values equal to or less than 15.06.

To ensure that the results yielded by the method are robust and consistent, we compare them to two other approaches using RFA: using only the animals that had survived the entire duration (140 days) of the original study and by looking at two other recovery threshold endpoints. First, the results of the RFA analysis using the subset of data are consistent with those of our analysis. On average, males took 33.58 days (s.e.=8.2 days) and females took 16.4 days (s.e.=1.45 days) to achieve 90% recovery. This leads to a difference of 17.19 days (95% CI 0.06, 34.32 days), thus also demonstrating that females reached 90% recovery more quickly than the males did (*P*=0.0494). The main difference in the results between our initial approach and this restricted data approach is the increased variability from the latter due to there being fewer animals (data points) for estimation and comparison. Next, to check for sensitivity to threshold choice, we performed the same analysis as presented above but changed the outcome threshold to 80% and 95%. For 80% recovery, on average, males took 19.62 days (s.e.=3.08 days) while females took 10.4 days (s.e.=1.03 days). On average, females reached 80% of their maximum recovery 9.21 days faster than males (95% CI 2.143, 16.28 days; *P*=0.01492). For 95% recovery, on average, males took 46.01 days (s.e.=11.47 days) while females took 22.32 days (s.e.=2.97 days). On average, females reached 95% of their maximum recovery 23.68 days faster than males (95% CI −2.13, 49.5 days; *P*=0.0688). The results from this dataset demonstrate that females are consistently recovering more quickly than males over a range of recovery thresholds.

The small sample sizes might suggest concern over using the *t* distribution; in particular, whether the distribution in the differences of the group means is approximately Normal. To examine this question, a Shapiro-Wilk Normality test was performed on the estimated mean days to recovery. The Shapiro-Wilk test results indicate that these data satisfy the Normality assumption (W=0.9304, *P*=0.3089).

## DISCUSSION

The purpose of this work was to demonstrate the RFA methodology in a biomedical research setting using previously published data as a working example. In the original study by Ghnenis et al. (2020), one of the questions of interest was if there was a sex-based difference in functional recovery at varying time points following SCI in rats. They found that, with the exception of a modest difference at a single early time point, there was no difference between male and female functional scores. A complimentary question, which was not investigated, is ‘is there a sex difference in the time it takes to recover from SCI?’, which we show how to answer here by demonstrating RFA's versatility and utility. These results, and the information provided by them, are in stark contrast to the comparison of overall recovery difference between the two sexes. When just comparing the two groups' average final recovery scores, there was no significant statistical nor functional difference for where each group ended: males and females each had a final average BBB score of about 10.

What differed between the two groups was not their landing points, but how quickly they got there, a question which repeated measures ANOVA cannot answer. Another downside to the more traditional repeated measures ANOVA is that it cannot use observations that drop out. In the context of the current example, that means that rats who died before the end of the study did not contribute data to the original analysis. This limitation can be overcome with RFA depending on the question being asked and the summary measure used. In this case, rats that survived until at least Day 28 (the time by which all animals' scores began to plateau) were included in the analysis, even if they subsequently died before the final time point. As mentioned earlier, spontaneous recovery begins to plateau starting around 4-6 weeks post injury, with the first 2 weeks post-injury generally being considered a ‘rapid recovery’ phase (Basso et al., 2006; Fedorova et al., 2022; McEwen and Springer, 2006). Therefore, the results RFA provides in showing that female rats reached 90% of their peak recovery more than 2 weeks (15.3 days) faster than males did, which is half the time it typically takes for recovery to plateau. These findings could be of great interest and encourage future research into the potential sexual dimorphism of functional recovery following SCI and potential reasons for it. This method enables more flexible data analysis as it has the potential to utilize more collected data and answer more questions than some of the most commonly used traditional methods allow.

## MATERIALS AND METHODS

### Experimental design

We start with a brief description of the of the original study by Ghnenis et al. (2020). The objective was to investigate the effects of SCI on non-nervous system tissue, as well as to assess any sex dependent responses to the injury. To assess the health-related aspects of SCI, cardio-metabolic and sensorimotor data were collected over the course of 20 weeks for the following variables: body composition; glucose and insulin metabolism; motor and sensory recovery; and echocardiography and histological evaluation of the heart. Adult male (*n*=10) and female (*n*=9) Sprague Dawley rats were used, and SCI was induced via contusion to the exposed spinal cord at the T10 level under anesthesia. Sham rats (*n*=6 per group), which underwent the same surgical process except for the actual SCI itself (no contusion), were used as a control. Due to attrition from the severity of the SCI, only five male and four female rats survived the entire 20 weeks of the study (see figure 1 of Ghnenis et al., 2020).

The statistical method outlined in this paper focuses on the evaluation of BBB scores from the rats that underwent SCI in the original experiment (there were no differences in the sham groups- all had consistently normal functional scores). The BBB rating scale is a 21-point scale originally developed for use in rat models of SCI, which discriminates phases of recovery based on injury severity and time after injury while possessing high sensitivity, intra-rater reliability, and validity (Basso et al., 1995, 1996a,b). Scoring was done during 4-min sessions by two independent observers watching the rats move in a small plastic pool and ranking hindlimb function on a predefined scale (Basso et al., 1996a; Mountney et al., 2013). These scores were then averaged to give a final score for each rat on each day testing was done. BBB testing was conducted twice pre-SCI (baseline testing) and then again post-injury at days 1, 4, 7, and then weekly thereafter for 20 weeks. BBB scores can range from 0 (paralyzed, no limb function) to 21 (normal, fully functional). The BBB results from Ghnenis et al. (2020), which were analyzed via repeated measures ANOVA, showed a significant difference between male and female scores only on Day 7 (average female BBB score was 4.44 points higher than male's, 95% CI 2.85, 6.03; *P*<0.001), with no significant difference in scores for the remaining days tested nor at the end of the experiment.

### Statistical method

The question we chose to answer was, ‘How long does it take for an animal to (essentially) recover?’. This question cannot be addressed by standard repeated measures approaches but can be answered by RFA. As we demonstrate here, RFA consists of a creative summary measure for each animal, followed by a formal statistical analysis on the summary measures.

**Fig. 2.**
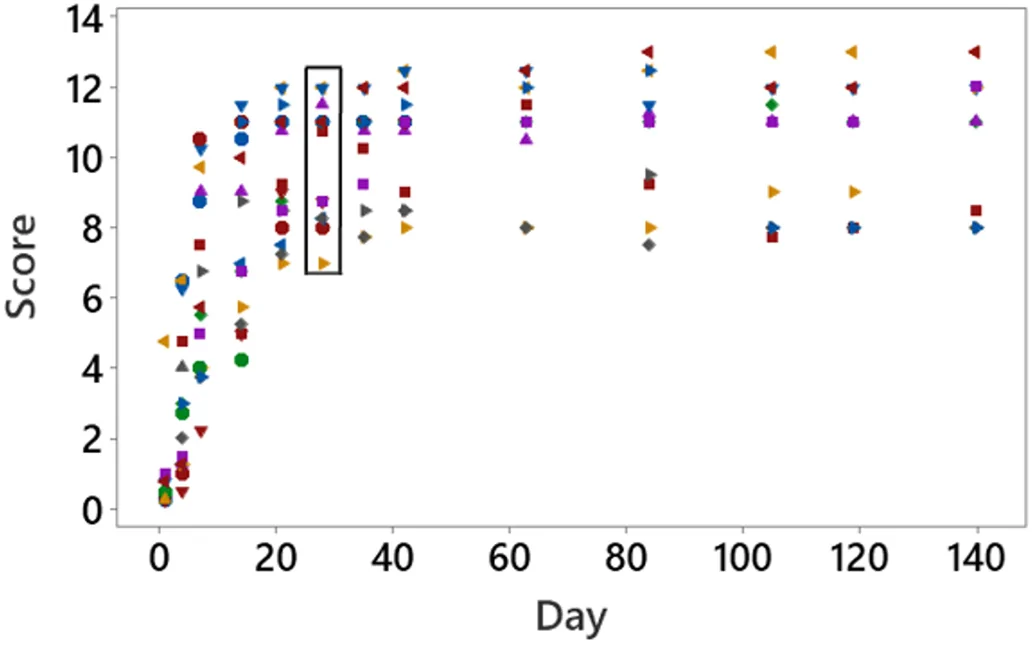
**BBB scores for all animals over time.** BBB scores for all the animals in the original study over the 20-week study period. Day 28 values are highlighted (black box) to show the time at which scores begin to approximately plateau.

First, the final recovery level was identified for each animal. To account for fluctuations in functional performance on test days, a trimmed mean, calculated using the animal's best three scores from Day 28 onward (when recovery began to plateau), was used as the final recovery value. Ultimately, recovery from many injuries is asymptotic; the last few percentage points of improvement can take a very long time. We will consider recovery, for humans, from hip/knee replacement or stroke as analogy to guide our thinking. Initial recovery can take place relatively quickly but the timeline can stretch out much further for ‘complete’ recovery and random good days and bad days can make it more difficult to gauge progress (Ballester et al., 2019; Lee et al., 2020; Zhang et al., 2026). In light of this, most people may feel that some large proportion of their total recovery would be nearly complete. For these reasons, we chose to use 90% of final recovery level as a focal point for our question and as our working definition of ‘essentially recovered’. We note that this choice is research question dependent, and RFA is not contingent on that choice. Fig. 2 shows that scores plateau around Day 35 and for most of the animals, 90% maximum recovery was reached by Day 28. This is consistent with previous literature which shows that, in rodent contusion models of spinal cord injury, spontaneous recovery beings to plateau around 4 to 6 weeks post injury (6 to 8 weeks for more severe injuries) (Basso et al., 2006; Fedorova et al., 2022; McEwen and Springer, 2006).

**Fig. 3.**
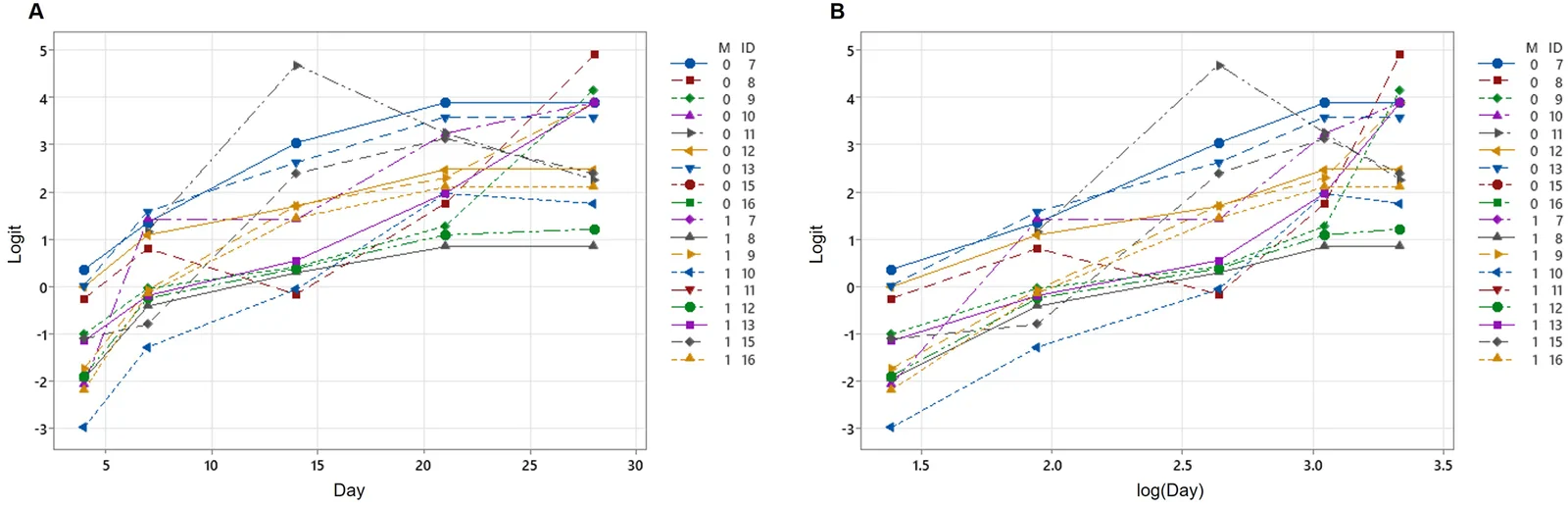
**Assessing linearity of relationship between score logits and time.** (A) Scatterplot of the BBB score logits versus day; curvature in the relationship can readily been seen. (B) Scatterplot of the BBB score logits versus log-transformed (using the natural log) day. After log-transformation, the relationship between the logits and time is approximately linear. The ID is the animal's identification number and M denotes whether the animal was male (M=1) or female (M=0).

Examination of the scores for each animal suggested that logistic regression provided a reasonable approach to obtain a useful summary measure. Accordingly, we rescaled the scores for each animal into a percentage of their recovery level. These percentages were very low at the start of the time sequence and approached their plateau level by Day 28. The logistic regression relationship worked best with time log-transformed, as the association between observed times (e.g. Day 4, Day 7, etc.) and logit(BBB scores) increased exponentially (Fig. 3A); upon transformation, the association between log(Time) and logit(BBB scores) became approximately linear (Fig. 3B).

**Fig. 4.**
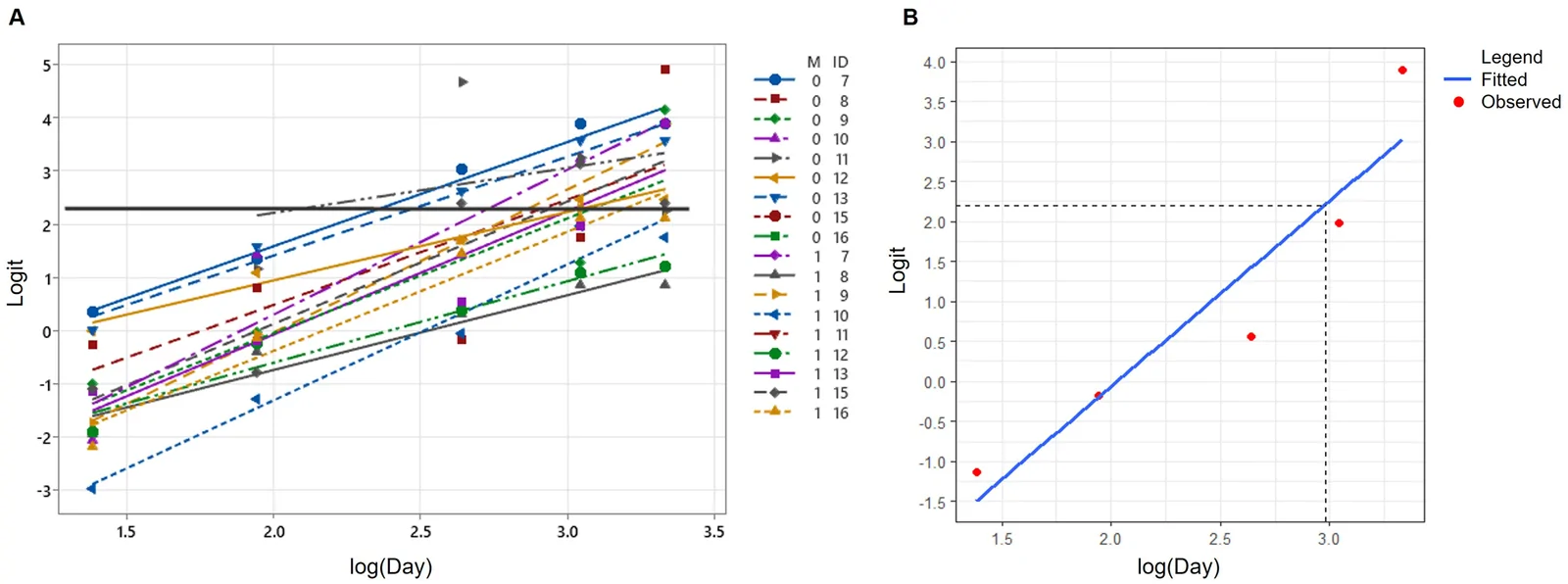
**Creative summary stage using logistic regression.** (A) Fitted regressions for all animals. The black horizontal line is set at *L*≈2.2, which corresponds to 90%. (B) Logits of recovery over time with fitted regression for one study animal (M13). The dashed horizontal line at *L*≈2.2 (90% recovery) intersects the fitted line at ln(Days)=2.98. The ID is the animal's identification number and M denotes whether the animal was male (M=1) or female (M=0).

Fig. 4A shows plots of the fitted regressions for all included animals. The black horizontal line at logit (*L*)≈2.2 corresponds to the 90% recovery threshold. Fig. 3B focuses on one of the study animals, where we show how ‘time to recovery’ was measured for that animal. A horizontal line set at 90% intersects the fitted line at ln(time)=2.98. That back-transforms to exp(2.98)=19.61 days. We repeated this process, arriving at recovery times for each animal.

## Supplementary Material


