# Supplementary material for: Response feature analyses for repeated measures behavioral data
Source: Biol Open. 2026 Aug 5;15(8):bio062488. doi: 10.1242/bio.062488 (PMC13488402; doi:10.1242/bio.062488)
Supplement: Supplementary file 1 [file biolopen-15-062488_review_history.pdf]

Response Feature Analyses for Repeated Measures Behavioral Data

Ken G. Gerow and Daniel T. Burns
10.1242/bio.062488

Editor: Valentina di Santo

Review timeline

Original submission:	13 January 2026
Editorial decision:	10 February
First revision received:	14 May 2026
Editorial decision:	28 May 2026
Second revision received:	2 July 2026
Editorial decision:	7 July 2026
Third revision received:	8 July 2026
Accepted:	13 July 2026

Original submission

First decision letter

MS ID#: bio.062488

MS Title: Response Feature Analyses for Repeated Measures Behavioral Data

Authors: Daniel T. Burns and Ken G. Gerow

I have now reached a decision on the above manuscript.

The reviewer reports are shown at the bottom of this email.

As you will see, the reviewers raised a number of substantial criticisms that prevent me from accepting the paper at this stage.

They suggest, however, that a revised version might prove acceptable, if you can address their concerns. If you think that you can deal satisfactorily with the criticisms on revision, I would be pleased to see a revised manuscript. We would then return it to the reviewers.

At this stage, we also ask you to ensure your manuscript complies with our formatting guidelines. Provided you are able to fully address the referees' comments, we are positive about publication of your paper (we accept over 95% of revision submissions) and therefore hope you won't mind any extra work involved in reformatting your manuscript at this point.

Please upload both a 'clean' version of your Word file, along with a highlighted version clearly showing where you have made changes in the revised manuscript. Please avoid using 'Track changes' in Word files as these are lost in PDF conversion.
I should be grateful if you would also provide a point-by-point response detailing how you have dealt with the points raised by the reviewers in the 'Response to Reviewers' box. Please attend to all of the reviewers' comments. If you do not agree with any of their criticisms or suggestions please explain clearly why this is so.

Reviewer 1

© 2026. Published by The Company of Biologists under the terms of the Creative Commons Attribution License (https://creativecommons.org/licenses/by/4.0/).

Comments for the author

The first two (or is it three?) paragraphs of the Introduction appear to be little more than a recitation of Jack did this with repeated measures and Jill did that. As written, these paragraphs could be beneficially deleted.

The summary of Ghnenis et al. contains a lot of detail that is extraneous to the analysis at hand, i.e., material that distracts from the objective for the current investigation. Communication in the present account would be improved by focusing on the BBB assessment of motor activity over time.

Specific comments:

Functional recovery was supposedly assessed for 6 males and 7 females over a period of 20 weeks (p. 5, line 7). Is this correct, or is this merely the number of rats that survived for 35 days?

I'm confused by what is meant by "observed times" (p. 5, line 9 ff). Does this mean that day of sampling (i.e., 1, 4, 7, 14, . . .) was log-transformed and that this transformation was the predictor in regressions?

"[T]he maximum recovery level for each animal was identified" (p.5, line 15). Does this mean that all the rats achieved their maximum by day 35 or did some of them achieve maximum after 35 days? This of concern because one male and one female seem to have died within the next week and could not be measured for potentially higher BBB scores (Fig. 1 in Ghnenis et al.). Does this mean that the last readings for the deceased rats were taken as their maxima? Or maybe the numbers provided in Figure 1 in Ghnenis et al. don't refer to animals that were available for assessments of BBB?

I don't understand the rationale for using a plateau in sequential BBB scores to identify maximum recovery for an animal when later scores are higher. Did the plateaus occur before day 35 or after? If the plateaus (or actual maxima) occurred after day 35, some rats already had died and could not be considered for higher maxima. How would the analysis be affected by using the actual maxima for animals having a plateau? Or by limiting analysis to data gathered up to, and including, day 35?

"[B]y Day 35 all animals had reached 90% of their maximum recovery" (p, 5, line 26 ff). How many animals that achieved maximum recovery were alive at the end of 20 weeks? Four females and five males? If so, maybe the analysis ought to be limited to those nine rats.

Also, all the animals had attained 90% recovery by day 35 (p. 5, line 26 ff), but two of the males were predicted not to reach 90% for over 40 days (Table 1). Is there a problem with this?

The original analysis (by Ghnenis et al.) or the first pass in our analysis (p. 5, line 32)?

Was the predictor "time" or "ln time" (p. 5, line 34)?

In the linear model (p. 5, line 48), is the predictor "X" or "ln X"?

Something is wrong with the handling and reporting of data by Ghnenis et al. Their figure 2 (which improperly shows equally spaced intervals along the X axis) presents means for BBB through 20 weeks. However, it looks like only nine animals survived to day 140. They seem also to have performed one repeated measures analysis on males, and another on females.

'Nuf said about Ghnenis et al. Let's look at how better to handle data for the current investigation. I recommend that the authors perform a repeated measures analysis for the combined data for males and females over the first 35 days. This is the interval for which full data are available for all animals. The authors should be able to tease out effects for sex (male vs. female), time (with its repeated measures on individual mice), and the time * sex interaction. Time would be expressed as days on a linear scale. The observations for day 4 may need to be omitted for reasons of interval along X axis (I will fudge for day 1). Results from this analysis should make for an interesting comparison with results of the new study by RFA.

© 2026. Published by The Company of Biologists under the terms of the Creative Commons Attribution License (https://creativecommons.org/licenses/by/4.0/).

The material in the paragraph beginning on page 5, line 32, really is pretty confusing. And what, exactly, is the point of the paragraph? It might be better here simply to focus on what was done and not muddy the water with alternatives.

How many decimal points are significant for reporting times, probabilities, and BBB scores?

<u>Reviewer 2</u>

Comments for the author

This manuscript is not ready for publication at its current level. Methodological contribution has merit, but significant deficiencies in presentation, methodological rigor, and statistical justification prevent acceptance in its present form.
Major concerns
The analysis relies on only 6 males and 7 females (n=13 total, reduced to n=12 after exclusion). This is underpowered for drawing meaningful conclusions about sex differences. The authors acknowledge a small sample size for bootstrap justification but fail to address power analysis or effect size considerations.
Why 90% recovery threshold specifically? Authors state it's "arbitrary" but provide no sensitivity analysis.
No assessment of model fit for individual animal regressions, no diagnostic plots or goodness-of-fit statistics, no discussion of whether linear regression on log-transformed data is appropriate for all 13 animals.
The authors claim RFA answers questions that "repeated measures ANOVA cannot answer" but never demonstrate what a mixed-effects model or growth curve analysis would show.
95% CI from bootstrap (4.34-19.59 days) is wider than t-test (1.75-21.44 days)—this discrepancy is not explained. The text reports different CIs in abstract (4.64-19.41) vs results (4.34-19.59)—which is correct?
No assessment of normality assumptions for t-test, no reporting of standard deviations, only means, no effect size measures (Cohen's d would be valuable).
The abstract claims females recovered "11.69 days earlier" (95% CI 4.64-19.41), but with n=6 males, one outlier could dramatically shift this estimate.
The biological significance of this difference is never established
RFA is acknowledged as existing since at least the 1990s, the only "new" contribution is applying it to this specific dataset, no theoretical advancement, simulation study, or comparison of RFA variants.
A simple histogram is insufficient. Should include, individual animal trajectories, fitted regression lines, residual diagnostics.

Reviewer's Responses to Questions

Experimental quality
Does each figure have the proper controls?
If 'No', please indicate reasons in Comments for Author box below.
Reviewer #1:
- Yes

Reviewer #2:
- No

Were the data analyzed using appropriate statistical tests?
If 'No', please indicate reasons in Comments for Author box below.
Reviewer #1:
- Yes

Reviewer #2:
- No

© 2026. Published by The Company of Biologists under the terms of the Creative Commons Attribution License (https://creativecommons.org/licenses/by/4.0/).

Reproducibility
Were experiments performed using adequate number of biological replicates?
If 'No', please indicate reasons in Comments for Author box below.
Reviewer #1:
- Yes

Reviewer #2:
- No

Does the methods section provide sufficient detail to permit reproducibility?
If 'No', please indicate reasons in Comments for Author box below.
Reviewer #1:
- Yes

Reviewer #2:
- No

Completeness
Are the manuscript's conclusions supported by the data?
If 'No', please indicate reasons in Comments for Author box below.
Reviewer #1:
- Yes

Reviewer #2:
- No

Scholarship
Do the authors cite and discuss the merits of data that would argue for and against their conclusion?
If 'No', please indicate reasons in Comments for Author box below.
Reviewer #1:
- Yes

Reviewer #2:
- No

Does the manuscript title & abstract accurately reflect the contents of the manuscript, without hyperbole?
If 'No', please indicate reasons in Comments for Author box below.
Reviewer #1:
- Yes

Reviewer #2:
- Yes

First revision

Author response to reviewers' comments

© 2026. Published by The Company of Biologists under the terms of the Creative Commons Attribution License (https://creativecommons.org/licenses/by/4.0/).

Dear Editor and Reviewers,

On behalf of my co-authors, I would like to thank you for the opportunity to revise our manuscript "Response Feature Analyses for Repeated Measures Behavioral Data." We sincerely appreciate the reviewers' time, thoughtful comments, and constructive suggestions. We do, however, want to make it abundantly clear that this work *is not* a biological study but rather a demonstration of a statistical method using previously published biological data as an example. The reviewer comments led us to think more deeply about certain details. The focus of our paper is on exemplifying RFA. Upon reflection, one step we took (reversing the role of the response (recovery score) and predictor (time), since we were trying to estimate time) is defensible theoretically, but in the end, we feel it added an unnecessary level of opacity. Readers trying to understand RFA might be put off if they think this step is key. We re-wrote the *Statistical Method* section without performing that step. The resulting explanation is

(1) Much cleaner to read and understand and
(2) Did not change the numerical results in any meaningful way.

Simpler is better, in this case. We have worked carefully to address each concern to the best of our ability and believe the manuscript has been significantly improved as a result.

Reviewer 1: General comments:

The first two (or is it three?) paragraphs of the Introduction appear to be little more than a recitation of Jack did this with repeated measures and Jill did that. As written, these paragraphs could be beneficially deleted.

The summary of Ghnenis et al. contains a lot of detail that is extraneous to the analysis at hand, i.e., material that distracts from the objective for the current investigation. Communication in the present account would be improved by focusing on the BBB assessment of motor activity over time.

RESPONSE: Thank you for this suggestion. We agree and have rewritten much of the Introduction to be more concise and relevant.

Specific comments:

Functional recovery was supposedly assessed for 6 males and 7 females over a period of 20 weeks (p. 5, line 7). Is this correct, or is this merely the number of rats that survived for 35 days?

RESPONSE: Thank you for pointing out this lack of clarification. In the original study by Ghnenis et al., 9 female and 10 male rats were initially used for the SCI group. Four males and two females died prior to day 35. By the end of the 20 weeks of that study, only 4 females and 5 males had survived (**Figure 1** in the Ghnenis paper). Six males and seven females were used/assessed in our present study demonstrating an application of the RFA method. More animals are included in our analysis than survived to the end of the Ghnenis study because our analysis only needs the animals to have survived to the point at which the animals reached at least 90% of their maximum score, which was 35 days for these data. We have gone through the manuscript to make it clearer the attrition that occurred in the Ghnenis study and how the number of animals assessed in our study differs, and why.

I'm confused by what is meant by "observed times" (p. 5, line 9 ff). Does this mean that day of sampling (i.e., 1, 4, 7, 14, . . .) was log-transformed and that this transformation was the predictor in regressions?

RESPONSE: Thank you for pointing this out and we apologize for the confusion. The "observed times" were the actual sampling days (1, 4, 7, etc.). In rewritten *Statistical Method* section, we have made it clear what the "observed times" are and why they needed to be log-transformed (the relationship between time and BBB scores was exponential rather than linear, but become linear upon log-transforming time).

© 2026. Published by The Company of Biologists under the terms of the Creative Commons Attribution License (https://creativecommons.org/licenses/by/4.0/).

"[T]he maximum recovery level for each animal was identified" (p.5, line 15). Does this mean that all the rats achieved their maximum by day 35 or did some of them achieve maximum after 35 days? This of concern because one male and one female seem to have died within the next week and could not be measured for potentially higher BBB scores (Fig. 1 in Ghnenis et al.). Does this mean that the last readings for the deceased rats were taken as their maxima? Or maybe the numbers provided in Figure 1 in Ghnenis et al. don't refer to animals that were available for assessments of BBB?

RESPONSE: Thank you for mentioning this. With the exception of the 6 animals that died before day 35, all other animals reached 90% of their maximum BBB scored by day 35. Of the 13 animals' data used in this study, two reached their maximum BBB score on day 35, the other 11 reached their maximums after day 35. **Figure 1** in the Ghnenis paper shows overviews in 4-week increments. All of the animals analyzed in the present study survived until at least Day 42; one male and one female did not survive past Day 42 and therefore their BBB data do not extend beyond that. However, both had the same BBB scores on Day 42 as they did on Day 35.

I don't understand the rationale for using a plateau in sequential BBB scores to identify maximum recovery for an animal when later scores are higher. Did the plateaus occur before day 35 or after? If the plateaus (or actual maxima) occurred after day 35, some rats already had died and could not be considered for higher maxima. How would the analysis be affected by using the actual maxima for animals having a plateau? Or by limiting analysis to data gathered up to, and including, day 35?

RESPONSE: Thank you. For the original approach that used the logits of the BBB scores, if you use the maximum BBB score then percent recovery would be 1 (100) and 1 minus percent recovery would be 0 and thus the entire approach would not work. The choice of recovery percentage is arbitrary depending on a researcher's needs/questions/background knowledge, so long as it doesn't result in having zero in the denominator (which 100% would do).
For the updated and simplified approach, recovery from an injury is asymptotic; the last few percentage points of improvement can take a very long time. Thus, we chose 90% for demonstration purposes as, with these data, the functional differences between 90% and 100% were negligible. None of the data collected after Day 35 was used in either version of the analysis we present.

"[B]y Day 35 all animals had reached 90% of their maximum recovery" (p, 5, line 26 ff). How many animals that achieved maximum recovery were alive at the end of 20 weeks? Four females and five males? If so, maybe the analysis ought to be limited to those nine rats.

RESPONSE: Thank you. Yes, only 4 females and 5 males survived the entire 20 weeks of the original study. Part of the utility of using RFA in this way for these data is that we don't have to exclude animals that didn't survive until the end of the 20 weeks like repeated measures ANOVA does; our method was able to analyze 13 animals from these data rather than only 9 as RMANOVA would.

Also, all the animals had attained 90% recovery by day 35 (p. 5, line 26 ff), but two of the males were predicted not to reach 90% for over 40 days (Table 1). Is there a problem with this?

RESPONSE: Thank you for mentioning this. This is not a problem as predictions are expected to be imperfect and variable compared to the actual observations; perfect prediction would indicate, at the very least, a very overfit model.

The original analysis (by Ghnenis et al.) or the first pass in our analysis (p. 5, line 32)?

RESPONSE: Thank you for pointing out this unclear wording. We have edited the sentence to now read "The first stage of this analysis…." to enhance clarity.

Was the predictor "time" or "ln time" (p. 5, line 34)?

RESPONSE: Thank you for this question. Time was the outcome, not the predictor. As mentioned previously, because "time" was not linearly associated with BBB score it was log transformed ("ln time"). The "ln time" estimates were calculated and then back-transformed (by exponentiating) to be regular "time" units in days.

© 2026. Published by The Company of Biologists under the terms of the Creative Commons Attribution License (https://creativecommons.org/licenses/by/4.0/).

In the linear model (p. 5, line 48), is the predictor "X" or "ln X"?

RESPONSE: Thank you. This should no longer be an issue as this revised version of the analysis has been updated to be simpler. However, to answer the question as it pertains to the original manuscript submission, the example shown there is of a general simple linear regression, thus the predictor is just X. For our study specifically, the predictor was the logit of the BBB score (ln(BBB)). As shown in Step 1 on pg.6, the BBB score logit is calculated by taking the log of the ratio of the percent of recovery over 1 minus the percent of recovery, for each time point measured.

Something is wrong with the handling and reporting of data by Ghnenis et al. Their figure 2 (which improperly shows equally spaced intervals along the X axis) presents means for BBB through 20 weeks. However, it looks like only nine animals survived to day 140. They seem also to have performed one repeated measures analysis on males, and another on females.

RESPONSE: Thank you for pointing this out. We can't comment on the perceived correctness of **Figure 2** other than to say that it had gone through the typical process to be published. You are correct that only 9 animals in total survived the entire 20 weeks of the original experiment (as seen in **Figure 1** of the Ghnenis paper).

'Nuf said about Ghnenis et al. Let's look at how better to handle data for the current investigation. I recommend that the authors perform a repeated measures analysis for the combined data for males and females over the first 35 days. This is the interval for which full data are available for all animals. The authors should be able to tease out effects for sex (male vs. female), time (with its repeated measures on individual mice), and the time * sex interaction. Time would be expressed as days on a linear scale. The observations for day 4 may need to be omitted for reasons of interval along X axis (I will fudge for day 1). Results from this analysis should make for an interesting comparison with results of the new study by RFA.

RESPONSE: Thank you for this comment. As seen in **Figure 1** of the Ghnenis paper, full data were not available over the first 35 days; by Week 4, 4 male rats and 1 female rat had died. We are not trying to redo the analysis from the original paper, we are comparing our RFA approach to the RMANOVA originally used to show a different, complimentary question that *could have* been answered with these data.

The material in the paragraph beginning on page 5, line 32, really is pretty confusing. And what, exactly, is the point of the paragraph? It might be better here simply to focus on what was done and not muddy the water with alternatives.

RESPONSE: Thank you for this comment. The *Statistical Method* section has been rewritten to show a simpler way (both in terms of understanding and doing) of carrying out our proposed analysis method.

How many decimal points are significant for reporting times, probabilities, and BBB scores?

RESPONSE: Thank you for this question. For reporting purposes, it depends on the norms of an individual field and/or a journal's reporting criteria. In our analysis, no rounding was performed until the very end when results were reported. Time (in days) is usually reported to one or two decimal places; for consistency, we have edited the manuscript so that all Time results are reported to one decimal place. BBB scores typically only go to two decimal places. For p-values, we tend to default to four decimal places unless instructed otherwise by journal requirements.

Reviewer 2: This manuscript is not ready for publication at its current level. Methodological contribution has merit, but significant deficiencies in presentation, methodological rigor, and statistical justification prevent acceptance in its present form.

Major concerns

The analysis relies on only 6 males and 7 females (n=13 total, reduced to n=12 after exclusion). This is underpowered for drawing meaningful conclusions about sex differences. The authors

© 2026. Published by The Company of Biologists under the terms of the Creative Commons Attribution License (https://creativecommons.org/licenses/by/4.0/).

acknowledge a small sample size for bootstrap justification but fail to address power analysis or effect size considerations.

RESPONSE: Thank you. We agree that the small sample of 13 (not 12) total animals is likely underpowered, but that is the data available from this previously published study. The purpose of the present study is not to draw meaningful conclusions about sex differences, or any biological variable(s), but rather to demonstrate a statistical method/approach for analyzing such data. For the purpose of this manuscript, power and effect size are irrelevant.

Why 90% recovery threshold specifically? Authors state it's "arbitrary" but provide no sensitivity analysis.

RESPONSE: Thank you for this comment. For the approach explained in our original submission, if you use the maximum BBB score, the percent recovery would be 100% and 1 minus percent recovery would then be 0 and thus the entire approach would not work (division by 0 is undefined). The choice of recovery percentage is arbitrary depending on a researcher's needs/questions/background knowledge, so long as it doesn't result in having zero in the denominator (which 100% would do). We chose 90% here for demonstration purposes, in the original and the revised method, as the 90% scores were functionally equivalent to the maximum scores. A sensitivity analysis is not needed as multiple modeling strategies are not being used to compare the same outcome. The choice of percent recovery is not made based on a rule or optimization for the modelling but rather driven by the research question (e.g., if a researcher was interested in the time it takes to get 50% of the way to some outcome, etc.). We have added more text to make this point clearer.

No assessment of model fit for individual animal regressions, no diagnostic plots or goodness-of-fit statistics, no discussion of whether linear regression on log-transformed data is appropriate for all 13 animals.

RESPONSE: Thank you. The initial step (individual animal regressions) is not a statistical analysis, per se. It uses a statistical tool as a measurement device. We examined the data graphically, and the tool we used was biologically reasonable. In RFA, formal statistical criteria come into play in the second stage, the summary measure stage is a measurement choice.

The authors claim RFA answers questions that "repeated measures ANOVA cannot answer" but never demonstrate what a mixed-effects model or growth curve analysis would show.

RESPONSE: Thank you for pointing this out. We do not demonstrate what those models show because 1) they are not the focus of our analysis and 2) just like RMANOVA, neither of those approaches can answer the question we use RFA to answer. Mixed effects models are more versatile compared to RMANOVA but would still be answering the same question(s), namely "is there a difference in recovery score between the two groups at each time point?" It does not answer the question "is there a difference in time until X% recovery and, if so, what is it?" We have added a discussion about these methods in the Introduction where we go over some of their benefits and shortcomings.

95% CI from bootstrap (4.34-19.59 days) is wider than t-test (1.75-21.44 days)—this discrepancy is not explained. The text reports different CIs in abstract (4.64-19.41) vs results (4.34-19.59)—which is correct?

RESPONSE: Thank you for pointing this out. T-test CIs are conservative estimates, especially in small sample contexts; this is why the estimate is larger than the CI obtained from bootstrapping. In the originally reported analysis, the female group reached 90% of their peak recovery, on average, 11.63 (95% CI 4.34-19.59) days earlier than did males. The abstract has been updated to show the correct values from the revised, simpler method we now show.

No assessment of normality assumptions for t-test, no reporting of standard deviations, only means, no effect size measures (Cohen's d would be valuable).

© 2026. Published by The Company of Biologists under the terms of the Creative Commons Attribution License (https://creativecommons.org/licenses/by/4.0/).

RESPONSE: Thank you. A t-test does not require Normality of the data. It *does* require approximate Normality of, in this case, the sample means. The Central Limit Theorem will assure us of this if the data are Normal enough. With large sample sizes, “enough” can be quite relaxed. Here, our small sample sizes do not give us that comfort and make assessing the data for Normality untrustworthy (with small samples, the distribution of the data can be wildly uninformative). We used bootstrapping and randomization testing, now reported at the end of the *Results* section, to assess Normality.

The abstract claims females recovered "11.69 days earlier" (95% CI 4.64-19.41), but with n = 6 males, one outlier could dramatically shift this estimate.

RESPONSE: Thank you. Yes, one outlier could shift the estimate. We introduced a fake 7th male (not included in the manuscript); its value would have to be 16.6 days or lower for the CI to include zero. We note that 16.6 is quite lower than the lowest observed male value (23.1). There are, however, 3 of 7 female values equal to or less than 16.6. That being said, the intent of our paper is to illustrate a method of answering questions that might be biologically interesting, not to say something in particular about this experiment.

The biological significance of this difference is never established

RESPONSE: Thank you. The purpose of the present study is solely to demonstrate a statistical method using previously gathered, analyzed, and reported data as an example. Potential biological significance is irrelevant for this purpose.

RFA is acknowledged as existing since at least the 1990s, the only "new" contribution is applying it to this specific dataset, no theoretical advancement, simulation study, or comparison of RFA variants.

RESPONSE: Thank you for this comment. RFA is an approach, not a singular method (e.g., like a t-test). It has existed for a few decades yet almost no one knows about it, especially not those outside of statistics. We feel that it is a scientifically powerful analysis method that deserves a brighter light shone on it. We have added text to the *Introduction* to make this point clearer to readers.

A simple histogram is insufficient. Should include, individual animal trajectories, fitted regression lines, residual diagnostics.

RESPONSE: Thank you. The summary measure was/is a measurement choice, not a statistical analysis per se. We are not interested in, for instance, whether the trajectory for any one animal is “statistically significant”. That would be of interest only if that animal was the entire universe of interest. The datum for each animal (days until recovery) is simply a measurement choice. However, with the simpler approach we now demonstrate in the revised submission, we have included a figure for the fitted logistic regression for one the study animals to demonstrate how the creative summary part was carried out. We have also added another figure to graphically show the estimated days to 90% recovery for each animal, as well as the sex-grouped mean recovery days (the data used for the statistical analysis part of RFA).

<u>Second decision letter</u>

MS ID#: bio.062488R1

MS Title: Response Feature Analyses for Repeated Measures Behavioral Data

Authors: Daniel T. Burns and Ken G. Gerow

I have now reached a decision on the above manuscript.

© 2026. Published by The Company of Biologists under the terms of the Creative Commons Attribution License (https://creativecommons.org/licenses/by/4.0/).

The reviewer reports are shown at the bottom of this email.

As you will see, the reviewers raised a number of substantial criticisms that prevent me from accepting the paper at this stage.

They suggest, however, that a revised version might prove acceptable, if you can address their concerns. If you think that you can deal satisfactorily with the criticisms on revision, I would be pleased to see a revised manuscript. We would then return it to the reviewers.

At this stage, we also ask you to ensure your manuscript complies with our [formatting guidelines]. Provided you are able to fully address the referees' comments, we are positive about publication of your paper (we accept over 95% of revision submissions) and therefore hope you won't mind any extra work involved in reformatting your manuscript at this point.

Please upload both a 'clean' version of your Word file, along with a highlighted version clearly showing where you have made changes in the revised manuscript. Please avoid using 'Track changes' in Word files as these are lost in PDF conversion.

I should be grateful if you would also provide a point-by-point response detailing how you have dealt with the points raised by the reviewers in the 'Response to Reviewers' box. Please attend to all of the reviewers' comments. If you do not agree with any of their criticisms or suggestions please explain clearly why this is so.

Reviewer 1

Comments for the author

The revised manuscript by Burns and Gerow is a substantial improvement over the original submission (which I examined as Referee #1). The revised account is shorter, more sharply focused, and much easier to read for comprehension. I now appreciate that the authors are merely using data from a published report to advocate for an underappreciated analytical procedure that handles data sets that cannot be examined by ANCOVA with repeated measures.

However, the authors chose a poor data set to illustrate the power and utility of the RFA protocol. By picking and choosing a subset of data for their analysis, I am left with the feeling that they have cherry-picked those data that provide the desired outcome for their analysis. The problem is what happened during the interval between days 35 and 42. Some animals didn't reach their plateau until after day 42 while others died before day 42. Two males apparently didn't attain a plateau in BBB until 6-7 weeks, but their performance is compared to that of two other mice that died after week 5.

So, I am ambivalent. The procedure that is presented as an alternative for repeated measures ANOVA is interesting and merits reporting in a forum like Biology Open. On the other hand, the treatment presented in the revised manuscript fails to convince me of the value for the protocol because of limitations of the data set. I'd suggest that the authors first analyze data for the nine mice that survived for the duration of the original study. If the outcome is unconvincing, I'd recommend that they look for a new data set.

Reviewer 2

Comments for the author

A methods demonstration paper still needs to meet basic standards of rigor. The good news is that most of the revisions needed here are not major undertakings, they are largely straightforward additions that would meaningfully strengthen the paper. Engaging with these points directly and with actual data, rather than arguing around them philosophically, would go a long way toward achieving acceptance.

Comment 1: Small sample size, no power analysis or effect size

© 2026. Published by The Company of Biologists under the terms of the Creative Commons Attribution License (https://creativecommons.org/licenses/by/4.0/).

Calling power and effect size "irrelevant" because this is a methods paper comes across as dismissive, and frankly, it is not a defensible position. The dataset chosen to demonstrate a method still must hold up to scrutiny, a demonstration is only as persuasive as the example behind it. Reporting Cohen's d for the difference in recovery times is a simple step that costs very little but adds real credibility. Similarly, a post-hoc power calculation is not about making biological claims, it is about helping readers understand whether the observed effect was realistically detectable given your sample size and what that tells them about the method's sensitivity in small-sample settings. That is directly relevant to anyone considering applying RFA to similarly sized datasets.

Comment 2: No sensitivity analysis for the 90% threshold
Stating a sensitivity analysis "is not needed" without any supporting rationale is not an argument. The 90% threshold is not a trivial choice, it effects every estimated recovery time in your analysis. Readers have no way of knowing whether your conclusions would hold at 80%, 85%, or 95%. Running those alternatives and showing the results are consistent would take modest effort and would actually serve the paper well, it would demonstrate that RFA is robust to reasonable variation in how the summary measure is defined, which is precisely the kind of flexibility you are trying to showcase.

Comment 3: No model fit assessment for individual animal regressions
The argument that per-animal regressions are just a "measurement choice" and therefore exempt from scrutiny does not really hold up. Whatever we call it, the regression fit for each animal is what generates the number that goes into t-test. If that fit is poor for even one or two animals, and with small, noisy biological data that is not an unreasonable worry, the downstream comparison is compromised. With only six or seven animals per group, there is no room to absorb that kind of error. What is needed here is fairly simple: report R^2 values for all 13 animals, and either include residual plots in a supplementary file or at least state clearly in the text that you examined the fits visually and they were satisfactory across the full sample. Showing one example animal in Figure 1 is a good start, but it is not sufficient evidence that the model worked well for everyone.

Comment 5: Discrepant confidence intervals between abstract and results
The response shifted into a discussion of t-test versus bootstrap differences, which only added confusion. To make matters worse, the revised manuscript now introduces a third set of values, so the problem has not been fixed, it has multiplied.

Comment 6: No standard deviations, no normality assessment, no effect size
Standard deviations for the per-animal recovery times are basic descriptive statistics — they should simply be in the table or results text. Their absence is noticeable. On the normality question, describing bootstrapping as a way to "assess normality" is not quite right, what bootstrapping does is provide robust inference about the mean difference, which is valuable, but it is not a normality test for the underlying data.

Comment 7: Sensitivity to a single outlier
The outlier robustness analysis you described in your response letter is actually a good idea, the problem is that it only exists in the letter not in the revised manuscript.

Comment 8: Biological significance never established
Nobody is asking to reframe the paper as a biology study. But if readers have no way of understanding whether a 14-day difference in recovery time actually matters in the context of rat spinal cord injury research, they cannot fully evaluate whether the method produced a useful answer. One or two sentences drawing on existing SCI literature to put that number in context would be entirely appropriate and would make the demonstration considerably more compelling, especially to the biomedical audience this journal serves. A methods paper is more citable and more impactful when readers can see that the method produced results that mean something.

Reviewer's Responses to Questions

Experimental quality
Are experiments performed using appropriate methods that will answer the question (or test the hypothesis or support the observations) posed by the authors? Is the right tool used for the job?
If 'No', please indicate reasons in Comments for Author box below.

© 2026. Published by The Company of Biologists under the terms of the Creative Commons Attribution License (https://creativecommons.org/licenses/by/4.0/).

Reviewer #1:
- Yes

Reviewer #2:
- No

Were the data analyzed using appropriate statistical tests?
If 'No', please indicate reasons in Comments for Author box below.
Reviewer #1:
- Yes

Reviewer #2:
- Yes

Reproducibility
Were experiments performed using adequate number of biological replicates?
If 'No', please indicate reasons in Comments for Author box below.
Reviewer #1:
- No

Reviewer #2:
- No

Is there sufficient raw data to assess the rigor of the analysis?
If 'No', please indicate reasons in Comments for Author box below.
Reviewer #1:
- No

Reviewer #2:
- No

Does the methods section provide sufficient detail to permit reproducibility?
If 'No', please indicate reasons in Comments for Author box below.
Reviewer #1:
- Yes

Reviewer #2:
- No

Completeness
Are the manuscript's conclusions supported by the data?
If 'No', please indicate reasons in Comments for Author box below.
Reviewer #1:
- No

Reviewer #2:
- No

Scholarship
Do the authors cite and discuss the merits of data that would argue for and against their conclusion?
If 'No', please indicate reasons in Comments for Author box below.
Reviewer #1:

© 2026. Published by The Company of Biologists under the terms of the Creative Commons Attribution License (https://creativecommons.org/licenses/by/4.0/).

- Yes

Reviewer #2:
- No

Does the manuscript title & abstract accurately reflect the contents of the manuscript, without hyperbole?
If 'No', please indicate reasons in Comments for Author box below.
Reviewer #1:
- Yes

Reviewer #2:
- Yes

Second revision

Author response to reviewers' comments

Dear Editor and Reviewers,

On behalf of my co-author, I would like to thank you again for the opportunity to revise our manuscript, "Response Feature Analyses for Repeated Measures Behavioral Data." We sincerely appreciate the reviewers' time, thoughtful comments, and constructive suggestions. We have worked carefully to address each concern to the best of our ability and believe the manuscript has been significantly improved as a result. Specifically, we have added multiple figures and revised previous figures to enhance transparency, data quality, and ease of understanding. Additionally, we have added a discussion based on current literature to put the use and results of our method into better practical context. To enhance the conceptual understanding of this process, we also changed how we calculated the maximum recovery values for each animal. Rather than simply selecting the largest value achieved by an animal, we used a trimmed mean, calculated from the top 3 values from each animal, to better account for the functional variability inherent in recovering from such injuries.

Comments from the Reviewers:

Reviewer 1: The revised manuscript by Burns and Gerow is a substantial improvement over the original submission (which I examined as Referee #1). The revised account is shorter, more sharply focused, and much easier to read for comprehension. I now appreciate that the authors are merely using data from a published report to advocate for an underappreciated analytical procedure that handles data sets that cannot be examined by ANCOVA with repeated measures.

However, the authors chose a poor data set to illustrate the power and utility of the RFA protocol. By picking and choosing a subset of data for their analysis, I am left with the feeling that they have cherry-picked those data that provide the desired outcome for their analysis. The problem is what happened during the interval between days 35 and 42. Some animals didn't reach their plateau until after day 42 while others died before day 42. Two males apparently didn't attain a plateau in BBB until 6-7 weeks, but their performance is compared to that of two other mice that died after week 5.

RESPONSE: Thank you. We respectfully disagree that this is a poor data set for showcasing the utility of RFA; to the contrary, we think it serves that purpose quite well. These data are a realistic dataset from a previously published study, that we had access to, which used a severe form of spinal cord injury. We hope that the addition of

© 2026. Published by The Company of Biologists under the terms of the Creative Commons Attribution License (https://creativecommons.org/licenses/by/4.0/).

the new **Figure 1** helps provide clarity for what happened to the animals in terms of their functional recovery throughout the duration of the original study. As explained in the manuscript, maximum (plateau) recovery values were not what was examined and compared between animals but rather 90% of that maximum. Thus, it only matters when an animal reached that 90% threshold, not the total maximum; animals reach this value at different times and that is what we want to compare. Further, once an animal has recovered, their scores would likely fluctuate from day to day, in the usual course of variation in life.

We have also added additional context and citations to the *Statistical Methods* section to show that the recovery trajectories of the animals from this experiment are consistent with what is seen in rodent models of spinal cord injury, chiefly that recovery plateaus around 4-8 weeks post-injury, depending on the severity. We were not looking for ways to eliminate animals—we sought to find legitimate ways to include *more* of them rather than discarding informative data.

So, I am ambivalent. The procedure that is presented as an alternative for repeated measures ANOVA is interesting and merits reporting in a forum like Biology Open. On the other hand, the treatment presented in the revised manuscript fails to convince me of the value for the protocol because of limitations of the data set. I'd suggest that the authors first analyze data for the nine mice that survived for the duration of the original study. If the outcome is unconvincing, I'd recommend that they look for a new data set.

RESPONSE: Thank you. To reiterate, one of the major points and draws of using RFA here is that we do not need to ignore these “extra” animals and only use the 9 that survived the entire study duration. We hope this is clearer now that we’ve added a new **Figure 1** showing the recovery trajectory of all of the animals where it is clear recovery started to plateau relatively early in the study for all animals (the boxed data at Day 28).

Reviewer 2: A methods demonstration paper still needs to meet basic standards of rigor. The good news is that most of the revisions needed here are not major undertakings, they are largely straightforward additions that would meaningfully strengthen the paper. Engaging with these points directly and with actual data, rather than arguing around them philosophically, would go a long way toward achieving acceptance.

Comment 1: Small sample size, no power analysis or effect size

Calling power and effect size "irrelevant" because this is a methods paper comes across as dismissive, and frankly, it is not a defensible position. The dataset chosen to demonstrate a method still must hold up to scrutiny, a demonstration is only as persuasive as the example behind it. Reporting Cohen's d for the difference in recovery times is a simple step that costs very little but adds real credibility. Similarly, a post-hoc power calculation is not about making biological claims, it is about helping readers understand whether the observed effect was realistically detectable given your sample size and what that tells them about the method's sensitivity in small-sample settings. That is directly relevant to anyone considering applying RFA to similarly sized datasets.

RESPONSE: Thank you. We have added the value of Cohen’s *d* to the Results section. We agree that a good example dataset is important for demonstrating a statistical method’s usefulness and utility. We believe that the additional details we have included in the manuscript text and through new figures demonstrate the adequacy of the data used here. However, we respectfully disagree regarding the utility of post-hoc power (PHP) analysis, both for this manuscript and any experimental setting in general. It has been well-documented in the literature over the last few decades that PHP analysis does not provide the information that those who use it think it does. Lenth (2001) showed that PHP is a function of the p-value of a test. Similarly, Hoenig and Heisey (2001) show the same conclusion—it just “repackages” the test’s p-value and adds no new information. Hoenig and Heisey (2001) also demonstrate that when shifting the PHP focus to

© 2026. Published by The Company of Biologists under the terms of the Creative Commons Attribution License (https://creativecommons.org/licenses/by/4.0/).

"detectable effect size" experiments with nonsignificant results further from the proposed null hypothesis value provide stronger evidence *for* the null hypothesis. Heinsberg and Weeks (2023) used simulation to demonstrate that PHP calculations are often misleading and uninformative for data interpretation and encourage authors and peer-reviewers to avoid using or requesting PHP calculations.

Lenth, R. V. (2001). Some practical guidelines for effective sample-size determination. The American Statistician, 55:187–193.

Hoenig, J. M. and Heisey, D. M. (2001). The abuse of power: The pervasive fallacy of power calculations in data analysis. The American Statistician, 55:19–24.

Heinsberg, L. W., & Weeks, D. E. (2022). Post hoc power is not informative. Genetic Epidemiology, 46, 390–394. https://doi.org/10.1002/gepi.22464

Comment 2: No sensitivity analysis for the 90% threshold

Stating a sensitivity analysis "is not needed" without any supporting rationale is not an argument. The 90% threshold is not a trivial choice, it effects every estimated recovery time in your analysis. Readers have no way of knowing whether your conclusions would hold at 80%, 85%, or 95%. Running those alternatives and showing the results are consistent would take modest effort and would actually serve the paper well, it would demonstrate that RFA is robust to reasonable variation in how the summary measure is defined, which is precisely the kind of flexibility you are trying to showcase.

RESPONSE: Thank you. We agree that the 90% threshold, while arbitrary, is not trivial. According to Kunselman (2023) and Thabane *et al.* (2013), sensitivity analysis strategies include examining outliers, distributional assumptions, and linear assumptions. In this revised manuscript, we have included the part of our prior response that explained the addition of a synthetic outlier to test model robustness, the results of Shapiro-Wilk Normality testing for distributional assumptions, and plots of the data (raw and fitted regressions) to assess linearity.

Kunselman, AR. A brief overview of sensitivity analyses. Fertility and Sterility 119, 6 (2023). https://doi.org/10.1016/j.fertnstert.2023.04.031

Thabane, L., Mbuagbaw, L., Zhang, S. et al. A tutorial on sensitivity analyses in clinical trials: the what, why, when and how. BMC Med Res Methodol 13, 92 (2013). https://doi.org/10.1186/1471-2288-13-92

Comment 3: No model fit assessment for individual animal regressions

The argument that per-animal regressions are just a "measurement choice" and therefore exempt from scrutiny does not really hold up. Whatever we call it, the regression fit for each animal is what generates the number that goes into t-test. If that fit is poor for even one or two animals, and with small, noisy biological data that is not an unreasonable worry, the downstream comparison is compromised. With only six or seven animals per group, there is no room to absorb that kind of error. What is needed here is fairly simple: report R^2 values for all 13 animals, and either include residual plots in a supplementary file or at least state clearly in the text that you examined the fits visually and they were satisfactory across the full sample. Showing one example animal in Figure 1 is a good start, but it is not sufficient evidence that the model worked well for everyone.

RESPONSE: Thank you. The newly included Figure 3 shows the fitted regressions plotted for all animals used in this study. We have also added text within the manuscript to clarify that the fits were analyzed and deemed satisfactory.

Comment 5: Discrepant confidence intervals between abstract and results

© 2026. Published by The Company of Biologists under the terms of the Creative Commons Attribution License (https://creativecommons.org/licenses/by/4.0/).

The response shifted into a discussion of t-test versus bootstrap differences, which only added confusion. To make matters worse, the revised manuscript now introduces a third set of values, so the problem has not been fixed, it has multiplied.

RESPONSE: Thank you for pointing this out. We have ensured that all reported values match and are correct throughout the manuscript. We primarily focus on the t-test results but mentioned bootstrapping and randomization in the *Results* to show that relying on Normality assumptions is okay for this small dataset. To further clarify, we have removed the brief discussion about randomization and bootstrapping and added the results of a Shapiro-Wilk Normality test to the end of the *Results* section which show that these data (the estimated mean days to recovery) are approximately Normal ($W = 0.9304$, $p = 0.3089$).

Comment 6: No standard deviations, no normality assessment, no effect size

Standard deviations for the per-animal recovery times are basic descriptive statistics — they should simply be in the table or results text. Their absence is noticeable. On the normality question, describing bootstrapping as a way to "assess normality" is not quite right, what bootstrapping does is provide robust inference about the mean difference, which is valuable, but it is not a normality test for the underlying data.

RESPONSE: Thank you. We have added the standard errors from the group comparisons to both the manuscript text and the updated Figure 4 showing the estimated per-animal and group mean times to 90% recovery. Normality testing on the underlying data should not be done as the underlying data are not expected nor assumed to be Normal. What needs to be approximately Normal is the distribution of the mean difference between the two groups.
Bootstrapping offers anecdotal evidence here in favor of Normality, as does the randomization test. We have added the results of a Shapiro-Wilk Normality test to the end of the *Results* section and removed the discussion about randomization and bootstrapping for reading ease and clarity.

Comment 7: Sensitivity to a single outlier

The outlier robustness analysis you described in your response letter is actually a good idea, the problem is that it only exists in the letter not in the revised manuscript.

RESPONSE: Thank you. We have added text to the manuscript similar to that provided in the previous round of review to explicitly state outlier robustness was assessed.

Comment 8: Biological significance never established

Nobody is asking to reframe the paper as a biology study. But if readers have no way of understanding whether a 14-day difference in recovery time actually matters in the context of rat spinal cord injury research, they cannot fully evaluate whether the method produced a useful answer. One or two sentences drawing on existing SCI literature to put that number in context would be entirely appropriate and would make the demonstration considerably more compelling, especially to the biomedical audience this journal serves. A methods paper is more citable and more impactful when readers can see that the method produced results that mean something.

RESPONSE: Thank you for this suggestion. We have added a discussion of these results based on existing SCI literature to the Discussion section to place them in a better context.

© 2026. Published by The Company of Biologists under the terms of the Creative Commons Attribution License (https://creativecommons.org/licenses/by/4.0/).

Third decision letter

MS ID#: bio.062488R2

MS Title: Response Feature Analyses for Repeated Measures Behavioral Data

Authors: Daniel T. Burns and Ken G. Gerow

Thanks for the revisions and for the care you've clearly put into responding to both reviewers. The paper is in noticeably better shape than the last round, and both reviewers say as much. That said, I think there are just a few minor things that still need attention before I can accept it.

Your response leaned on general sensitivity-analysis literature: outliers, normality, linearity, but that wasn't really what was asked. The reviewer wanted to see the actual recovery-time estimates recalculated at, say, 80% and 95% instead of 90%, just to confirm the sex difference doesn't disappear or flip depending on that choice. A short paragraph or small table showing the alternate numbers would be great.

Reviewer 1 asked that you show what the analysis looks like using only the 9 animals that made it the whole 20 weeks, as a way of addressing the cherry-picking concern head-on. Your response explains why you included the animals that died early, which is reasonable, but it doesn't actually show that comparison.

One small thing: the Heinsberg & Weeks citation is dated 2023 in your response letter but 2022 in the reference list. Just needs to be consistent.

Third revision

Author response to reviewers' comments

We have addressed the below-mentioned issues from the prior round of peer review by adding a paragraph to the *Results* section in which we discuss the results from the additional analyses. Specifically, the results from the sensitivity analysis are consistent with the results from our initial 90% choice (both 80% and 95% show females recovering more quickly than males) and the results from the restricted data set analysis are also the same as those from our initial approach, just with more variability due to the smaller sample size.

Fourth decision letter

MS ID#: bio.062488R3

MS Title: Response Feature Analyses for Repeated Measures Behavioral Data

Authors: Daniel T. Burns and Ken G. Gerow

I am happy to tell you that your manuscript has been accepted for publication in Biology Open, pending our standard publication integrity checks. It was accepted on 13th July 2026.

© 2026. Published by The Company of Biologists under the terms of the Creative Commons Attribution License (https://creativecommons.org/licenses/by/4.0/).
